# A case of cutaneous toxigenic *Corynebacterium ulcerans* likely acquired from a domestic dog

**DOI:** 10.1099/acmi.0.000025

**Published:** 2019-05-30

**Authors:** Richard Othieno, Kate Mark, Michelle Etherson, Geoffrey Foster, Steven Murray, Pota Kalima, Norman K. Fry, Claire Cameron, Jenni Strachan

**Affiliations:** ^1^​ National Health Services (NHS) Lothian, Edinburgh, UK; ^2^​ Scottish Agricultural College, Inverness, UK; ^3^​ Public Health England, London, UK; ^4^​ Health Protection Scotland, Glasgow, UK

**Keywords:** *Corynebacterium*, wound infection, zoonoses, public health, vaccination

## Abstract

**Introduction:**

*
Corynebacterium ulcerans* can produce diphtheria toxin and although still rare, is now the predominant cause of toxigenic diphtheria infection in the UK, making this organism of great clinical and public health importance. Here we describe a cutaneous case, likely secondary to domestic animal contact.

**Case presentation:**

A 60-year-old female presented with a slow-healing finger-burn wound. A skin swab cultured *Corynebacterium ulcerans,* which was confirmed to be toxin producing. She resided with her partner and two dogs, one of which had a chronic skin lesion. Her most recent diphtheria vaccine was in 2009. Four close contacts were identified, two of whom were healthcare professionals, and nose and throat swabs were obtained. The patient was treated with clarithromycin (14 day course), diphtheria vaccine and excluded from work until completion of antibiotics and negative clearance swabs. Contacts were given erythromycin (7 day course), vaccinated and healthcare worker contacts excluded from work until swab negative. A veterinary practitioner swabbed the throats and a skin lesion of their dogs. One contact (partner of patient) and all dog swabs were positive. Partial allelic profiles from MLST supported an epidemiological link. The dogs were treated with antibiotics and antimicrobial skin wash. Repeat swabs for the index case, contact and both dogs were negative following treatment.

**Conclusion:**

This was a rare case of cutaneous diphtheria secondary to *Corynebacterium ulcerans* with domestic animals the most likely source, although human-to-human contact could not be excluded, with important human and animal public health implications.

## Introduction

*Corynebacterium ulcerans* is an aerobic Gram-positive bacillus, which is capable of producing diphtheria exotoxin making identification of this organism of great clinical and public health importance. It is a zoonotic infection, historically associated with cattle, and other domestic animals including cats and dogs [1, 2]. *Corynebacterium ulcerans* has also been recovered from wild animals, including otters in Scotland and England [3]. Infection with *C. ulcerans* can cause the clinical syndromes of respiratory or cutaneous diphtheria. Cases of diphtheria may present with skin lesions that are indicative of cutaneous diphtheria, or respiratory symptoms associated with pseudo-membranes covering the trachea or bronchi [4, 5].

Human infection with *C. ulcerans* can be fatal and four deaths have occurred in the UK between 1986 and 2014 [6]. Although extremely rare, with 11 cases of diphtheria caused by *C. ulcerans* occurring in Europe in 2012 [7], the frequency and severity of infections associated with *C. ulcerans* appears to be increasing [2]. In addition, *C. ulcerans* has been the predominant cause of toxigenic infection in the UK since the 1990s, when *C. diphtheriae* was more common [8]. The reasons for this change in epidemiology is not clear, but may be related to pet ownership, as it is estimated that half of all UK households own a pet [9]. The majority of cases of *C. ulcerans* associated with zoonotic infection occurred in adults who had been partially or fully vaccinated with diphtheria toxoid [10]. It usually occurs in humans with a history of close animal contact, although previously it was thought that *C. ulcerans* was acquired from contact with cattle or consumption of raw dairy products, in more recent years, cases are increasingly associated with companion animals [4, 5, 11, 12]. A number of other species have been identified as carriers, including domestic pets and the pathogen has been identified in healthy dogs in urban areas of a number of countries [10, 13]. In the UK, there have been few cases of documented cutaneous toxigenic *C. ulcerans* infection associated with domestic animals [14] and [15]. Human–human transmission of toxigenic *C. ulcerans* is rare with only one report of a respiratory case documented [16]. Between 1986 and 2008 in the UK, *C. ulcerans* was identified in asymptomatic carriers of two separate cases of respiratory diphtheria [17]. There have been no documented cases of human–human transmission of *C. ulcerans* with a cutaneous presentation. Here we present a case of *C. ulcerans* skin infection due to domestic animal contact.

## Case report

In 2017, a 60-year-old female presented to her General Practitioner (GP) in Scotland with a slow-healing 1 cm wound on her right finger. She had sustained a minor burn to the finger 9 days prior to her attendance. A swab was taken by the attending clinician. This swab subsequently grew toxigenic *C. ulcerans *. This result was telephoned to the out-of-hours local health protection team, 8 days after the case presented to her GP.

The case lived with her partner and owned two German Shepherd dogs. One dog had a history of a long-standing skin complaint, which had been documented as quiescent. The case worked in retail and had no recent travel history. She had not eaten unpasteurized dairy food nor visited any farms within the last 2 weeks. Her most recent diphtheria vaccination was in 2009.

A nose and throat swab were collected from the patient and she was treated with diphtheria vaccine and a 2 week course of clarithromycin. She was excluded from work under the Public Health Act Scotland (2009) until completion of antibiotics and clearance swabs, one from each site (nose, throat and wound) were negative. Four close contacts were identified who required nose and throat swabs to assess for carriage of *C. ulcerans *, of whom two were healthcare professionals who had dressed the wound. Contacts were all given diphtheria vaccine and a 1 week course of erythromycin. The two healthcare workers were excluded from work until nose and throat swabs were culture negative. One contact (husband) was identified as carrying *C. ulcerans* in his nose and required a repeat swab following completion of antibiotics to check for clearance. His repeat swab was culture negative. Initial swabs for the fourth contact (a close friend) were negative.

A local veterinary practitioner examined the dogs, which revealed one had an infected skin lesion, which was swabbed. Throat swabs were also collected from both dogs. Toxigenic *C. ulcerans* was recovered in culture from the skin lesion and throat swabs of both dogs. They were commenced on antibiotic therapy and antimicrobial skin wash. Repeat swabs for the dogs were negative following completion of treatment. The dogs were identified as the most likely source of infection, however, it is possible human–human transmission may have occurred.

### Microbiology investigations

Wound swab was submitted for routine culture and was inoculated on Columbia blood agar (Thermo-Fisher, Perth, UK). Large numbers of *C.* *ulcerans* were cultured and identified using MALDI-TOF (MALDI Biotyper, Bruker, Massachusetts, USA). MALDI-TOF generates unique mass spectrometry profiles, which are compared to a known database of micro-organism profiles, identifying the organism to genus and species levels [18]. The MALDI-TOF score was >2.0, which is an acceptable score for species identification. Susceptibility testing was performed by E-test (bioMérieux) using European Society of Clinical Microbiology and Infectious Disease (EUCAST) interpretative criteria. The isolate was sent to the Diphtheria National Reference Laboratory, Public Health England (PHE), Colindale, London, where the isolates were characterized by genotypic and phenotypic methods. In April 2014, a real-time PCR (qPCR) assay was formally introduced as the front-line test for putative toxigenic corynebacteria to inform public health action [19]. This assay provides confirmation of both identification of *C. diphtheriae* and *C. ulcerans */* C. pseudotuberculosis* and detection of the diphtheria toxin gene. Phenotypic characterization was perfomed by culture on Columbia horse blood, Hoyle’s tellurite and Tinsdale agar plates (PHE Media Services, Colindale); API Coryne (bioMerieux) and additional differential biochemical tests (e.g. nitrate reduction, glycogen hydrolysis as required [20]. The modified Elek immunodiffusion test [21] was used to confirm toxin expression. Further genotypic characterization of isolates was performed by MLST as previously described [22, 23]. The clinical isolate from the index case was identified as *C. ulcerans */*C.diphtheriae,* diphtheria toxin gene positive by qPCR. The species was confirmed as *C. ulcerans* phenotypically and toxin expression was confirmed by the Elek test. Antimicrobial susceptibility testing results were also confirmed at the reference laboratory and it showed resistance to penicillin and clindamycin but sensitivity to vancomycin, erythromycin, linezolid, ciprofloxacin, doxycycline and rifampicin. *S. aureus* was isolated along with *C. ulcerans. S aureus.* could be colonizing the skin but was also likely contributing to any skin and soft tissue infection.

*C. ulcerans* was also isolated from a nose swab from the close contact of the index case and this was confirmed as toxigenic *C. ulcerans* as above. Throat swabs from the two dogs and the skin lesion were collected by their veterinary practitioner and submitted to SAC Consulting Veterinary Services laboratory in Inverness where they were cultured on Columbia sheep blood agar and Hoyles tellurite medium (Thermo-Fisher, Perth, UK). *C. ulcerans* was obtained in moderate growth from the throat swabs collected from each dog and in heavy growth from the wound. The wound also contained a heavy growth of *Staphylococcus schleiferi* subsp. coagulans*,* and an unidentified Gram-positive coccus. Suspect *C. ulcerans* were identified with the API Coryne system. Follow-up samples were collected from the throats of both dogs after treatment was completed and *C. ulcerans* was not detected. Furthermore, the skin lesion had resolved and neither *C. ulcerans *, *S. schleiferi subsp. coagulans* nor the unidentified Gram-positive coccus were detected. The three canine isolates from the two dogs were also confirmed as toxigenic *C. ulcerans *.

The six isolates (three human, three canine) were subjected to MLST analysis. Full profiles were not obtained, most likely due to variation in primer binding sites. However, of the partial alleleic profiles obtained (range 2 to 5 out of 7 alleles); all alleles occurring in >1 isolate matched and there were no mismatches, supporting an epidemiological link. All partial allelic profiles obtained (0, 41, 79, 49, 0, 45, 39) were consistent with sequence type 349 (42, 41, 79, 49, 49, 45, 39), which is present in the MLST database (https://pubmlst.org/cdiphtheriae/) [24], from *C. ulcerans* isolated from a cutaneous clinical case in 2005, from Toulouse, France.

## Discussion

Due to the rarity of this case, a literature review was carried out to identify relevant evidence of *C. ulcerans* and domestic animals. MEDLINE was searched for combinations of the terms ‘*Corynebacterium ulcerans*’, ‘diphtheria’, ‘zoonoses’ and ‘human’, limited to English only, from 1966 to date. Eight individual case reports were identified. The study characteristics are summarized in Table 1. Three of the eight cases involved dogs, and of these, one had cutaneous presentation. Our case was distinct from any of those identified in the literature for several reasons. Within the household, the case presented with cutaneous diphtheria and an asymptomatic carrier was also identified. This may represent the first case of human–human transmission of *C. ulcerans* in a cutaneous case. However, we acknowledge that both human infections may have been acquired from the dogs. In addition, our case was normally healthy with a history of minor trauma causing a wound. In other reported cases, the patients had a history of chronic illness that may have made them more susceptible to infection. The apparent increasing incidence of *C. ulcerans *, and the potential of the microbe to cause infection beyond the opportunistic spectrum suggests that this potentially deadly infection requires increased vigilance from public health.

**Table 1.** Summary of clinical features and transmission data of *C. ulcerans* infections from case reports from 1966–present (English language only)

**Reference**	**Transmission**	**Clinical presentation**	**Other features**
[26]	Dog–human	Severe dyspnoea	Case was immunosuppressed
[27]	Dog–human	Sore throat	Case died
[28]	Cat–human	Sore throat	Case had rheumatoid arthritis
[29]	Cat–human	Skin	Case previously well
[30]	Cat–human	Skin	Case died
[31]	Cat–human	Skin	Case was immunosuppressed
[16]	Human–human	Sore throat	Domestic cat in household
[32]	Dog – human	Skin	Case had history of chronic venous insufficiency
[14]	Cats, dogs, fox–human	Skin	Case previously well

### Implications for public health

The number of cases of diphtheria caused by toxigenic *C. ulcerans* with an epidemiological link to domestic animals is small, but rising [8]. Several deaths have been associated with *C. ulcerans *, both within the UK, and outside [7]. This case highlighted some of the challenges of managing diphtheria that could have significant impact on future management if cases continue to rise. Due to the rarity of this condition and the widespread diphtheria vaccination programme, recognition and diagnosis of cutaneous diphtheria may be delayed. This can lead to delay in treatment of the case, but also contact tracing and identification of a potential source.

The evidence base for the risk factors for acquisition of *C. ulcerans* is limited. The association with domestic companion animals who carry the organism is becoming increasingly apparent. However, although cases of *C. ulcerans* may have a history of contact with domestic pets, microbiological evidence of a link is often unavailable as the animals have only been investigated in more recent years [17]. Our case highlights the importance of identifying the source through epidemiological and microbiological investigation. Currently, national guidance for the management of diphtheria is available in the UK, provided by Public Health England [8]. However, this guidance does not include management of animal contacts. *C. ulcerans* is not a notifiable organism if detected in animals, including those implicated in human infection and guidance relating to the management of animals in a case such as this is not readily available. This is a potential area for development as the evidence base for this infection continues to expand.

Due to the multiagency management required in this case, we recommend that both human and animal public health is considered in the investigation and management of cases of *C. ulcerans* in the future. This is consistent with the One Health approach [25]. The complexities of case management that need to be addressed, include the following: assessing the risk in the individual, the public and the animal population; management of exclusion of cases, contacts and pets; and clinical and financial responsibilities of agencies in the assessment, investigation and treatment of cases, contacts and domestic companions. Through a co-ordinated approach, further research should be conducted into the prevention and management of this increasingly prevalent and potentially deadly infection, acknowledging the threat to animals and humans.
